# Usefulness of Implantation of Diffractive Multifocal Intraocular Lens in Eyes with Long Axial Lengths

**DOI:** 10.1155/2015/956046

**Published:** 2015-11-01

**Authors:** Tomoichiro Ogawa, Takuya Shiba, Hiroshi Tsuneoka

**Affiliations:** Department of Ophthalmology, The Jikei University School of Medicine, 3-25-8 Nishishinbashi, Minato-ku, Tokyo 105-0003, Japan

## Abstract

*Purpose*. This study retrospectively analyzed the postoperative visual functions of myopic eyes implanted with multifocal intraocular lens (IOL) to evaluate the efficacy of multifocal IOL in highly myopic eyes. *Methods*. We studied 61 patients (96 eyes) who were implanted with multifocal IOL ZMA00 or ZMB00 (Abbott Medical Optics). The patients were stratified into two groups by axial length: 26 mm or above (AL ≥ 26 group) and below 26 mm (AL < 26 group). Postoperative corrected and uncorrected distance (5 m) and near (30 cm) visual acuity (VA), contrast sensitivity, and depth of focus were compared between two groups. *Results*. In the AL ≥ 26 group and the AL < 26 group, the mean ± standard deviation uncorrected distance logMAR VA at 12-month postoperative follow-up was −0.04 ± 0.11 and −0.01 ± 0.14, respectively; and the corrected distance VA was −0.17 ± 0.08 and −0.14 ± 0.07, with no significant differences between two groups (*p* = 0.558 and 0.101; Mann-Whitney *U* test). For near VA, the corresponding uncorrected VA was 0.06 ± 0.08 and 0.05 ± 0.09; and distance-corrected VA was 0.01 ± 0.06 and 0.01 ± 0.02, with no significant differences between two groups (*p* = 0.572, and 0.157; Mann-Whitney *U* test). *Conclusion*. The present study demonstrates that it is possible to achieve good uncorrected near and distance VA following implantation of multifocal IOL in eyes with long axial lengths.

## 1. Introduction

High myopia is a risk factor of cataract [1–3]. The risk of developing cataract is higher at relatively younger ages, and the proportion of high myopic eyes in all cataract surgeries is substantially high. Recent studies have shown good clinical outcome of implantation of multifocal intraocular lens (IOL) following cataract surgery, and the evaluations also included a considerable number of high myopic eyes implanted with multifocal IOL. In young persons, cataract usually involves one eye only. In these cases, one eye undergoes cataract surgery, while the contralateral eye remains phakic. Implantation of a monofocal IOL will result in loss of accommodation ability in one eye, which may impair postoperative visual function. Therefore, implantation of multifocal IOL with a wide zone of clear vision is probably a useful option. Moreover, for patients with high myopia who are highly dependent on spectacles and contact lenses, multifocal IOL implantation is expected to reduce the dependence on spectacles and contact lens and increase patient satisfaction.

However, high myopia may be associated with various complications such as myopic macular lesion and myopic optic neuropathy. These conditions may affect visual function and may lead to hesitation over multifocal IOL implantation.

We performed a retrospective analysis of postoperative visual function of high myopic patients who underwent multifocal IOL implantation, with the aim to evaluate the usefulness of multifocal IOL in high myopic eyes.

## 2. Patients and Methods 

### 2.1. Patients

Sixty-one patients (96 eyes) aged 40 years or above who underwent multifocal IOL implantation at the Jikei University Hospital between 2009 and 2014 and were followed up for at least 12 months after surgery were studied. The subjects comprised 28 males (41 eyes) and 33 females (55 eyes) with a mean age of 59 years (range 40 to 71 years). Axial length was measured before surgery, using an IOLMaster version 5 or IOLMaster 500 (Carl Zeiss Meditec). Since long axial length is generally defined as axial length exceeding 26-27 mm, the patients were stratified into two groups according to the preoperative axial length: 26 mm or above (AL ≥ 26 group: 21 patients, 32 eyes) and below 26 mm (AL < 26 group: 40 patients, 64 eyes). There was no difference in age between the two groups (*p* = 0.703). Patient characteristics of the two groups are shown in Table 1.

The criterion for preoperative corneal astigmatism was −1.50 D or below. Before surgery, ophthalmoscopy and optical coherence tomography (OCT) were conducted. Patients with definitive macular disease or glaucoma, those with ocular diseases likely to affect postoperative visual function, and those with severe intraoperative or postoperative complications were excluded from the study.

The present study was approved as a retrospective study by the Ethical Committee of the Jikei University Hospital.

### 2.2. Methods

The IOL used were ZMA00 and ZMB00 (Abbott Medical Optics) that have the same optical design. The surgeries were performed by three ophthalmologists. From a 2.4-mm temporal corneal incision or superior sclerocorneal incision, cataract was removed by phacoemulsification and aspiration. Then the IOL was implanted inside the lens capsule using an injector. For the selection of IOL power, hyperopia relative to the predicted refractive power was presumed to occur in the AL ≥ 26 group. Each operator chose the target refractive power based on experience and selected the IOL power aiming to obtain postoperative refraction of 0 D.

At 1 week, 1 month, 2 months, 3 months, 6 months, and 12 months after surgery, uncorrected and corrected distance (5 m) and near (30 cm) visual acuity were measured. At 1 month after surgery, OCT examination was performed using a Cirrus OCT (Carl Zeiss Meditec). At 3 months after surgery, contrast sensitivity was measured using a Takagi Glare Tester CGT-1000 (Takagi Seiko Co. Ltd.), and depth of focus was also determined. To generate the defocus curve, first the patient was corrected for best distance visual acuity (using distance full correction lens) and the power was normalized to 0. Then additional lens was added to introduce defocus from spherical equivalent of +2.0 to −5.0 D in 0.5 D increments, and the corrected visual acuity was measured on a 5-m visual acuity chart after each increment. Results of the 15 tests were used to plot the defocus curve. In addition, the patients were interviewed regarding the status of spectacle or contact lens use after surgery and for the level of satisfaction after multifocal IOL implantation.

The clinical results of the two groups were compared using the Mann-Whitney *U* test. Postoperative spherical equivalent and rate of spectacle use after surgery were compared between two groups using chi-squared test. A *p* value less than 0.05 was considered to indicate statistical significance.

## 3. Results 

### 3.1. Visual Acuity

**Figure 1:**
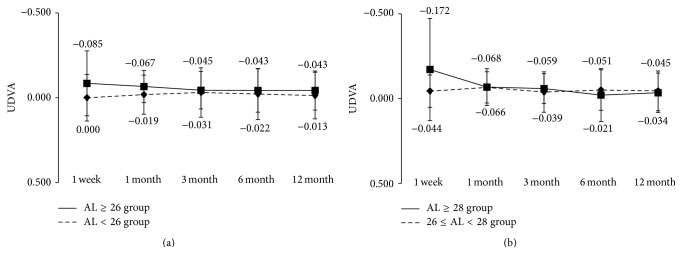
Uncorrected distance visual acuity during 12-month follow-up after implantation of multifocal intraocular lens. (a) Eyes were divided into axial length of 26 mm or longer (AL ≥ 26 group) and less than 26 mm (AL < 26 group). (b) Eyes in AL ≥ 26 group were further divided into 26 ≤ AL < 28 group and AL ≥ 28 group. ^*∗*^NS Mann-Whitney *U* test.

**Figure 2:**
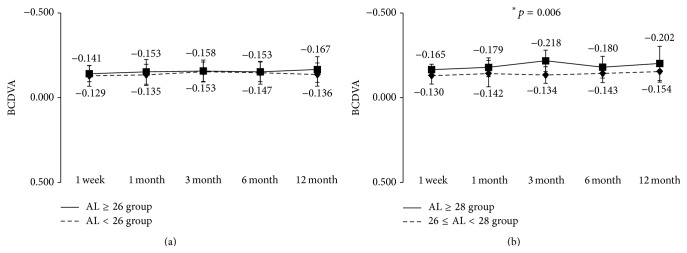
Best corrected distance visual acuity during 12-month follow-up after implantation of multifocal intraocular lens. (a) Eyes were divided into axial length of 26 mm or longer (AL ≥ 26 group) and less than 26 mm (AL < 26 group). (b) Eyes in AL ≥ 26 group were further divided into 26 ≤ AL < 28 group and AL ≥ 28 group. ^*∗*^Mann-Whitney *U* test.

The results of visual acuity in the AL ≥ 26 and AL < 26 groups are shown in Figures 1(a) and 2(a). Visual acuity is expressed as mean ± standard deviation (SD) of logarithmic minimum angle of resolution (logMAR).

At 12 months after surgery, the mean uncorrected distance visual acuity in the AL ≥ 26 group and the AL < 26 group was −0.04 ± 0.11 and −0.01 ± 0.14, respectively (Figure 1(a)), and the mean corrected visual acuity was −0.17 ± 0.08 and −0.14 ± 0.07 (Figure 2(a)). During the observation period from 1 week to 12 months after surgery, both uncorrected and corrected visual acuity were not significantly different between the two groups. Good visual acuity was achieved early after surgery in both groups, with uncorrected visual acuity of 0.10 or above and corrected visual acuity of 0.0 or above throughout the observation period. We also divided the AL ≥ 26 group into a group with 26 ≤ AL < 28 and a group with AL ≥ 28 mm and conducted the same analysis. Apart from a significant difference in corrected distance visual acuity at 3 months after surgery, no other significant differences were found between two groups (Figures 1(b) and 2(b)).

**Figure 3:**
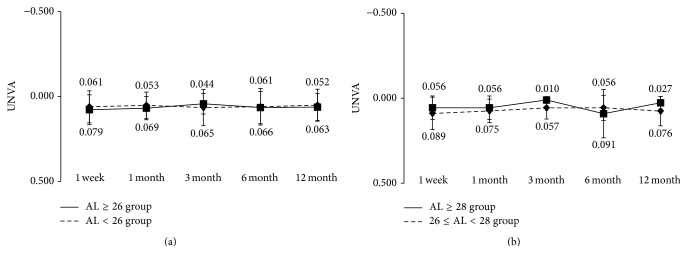
Uncorrected near visual acuity during 12-month follow-up after implantation of multifocal intraocular lens. (a) Eyes were divided into axial length of 26 mm or longer (AL ≥ 26 group) and less than 26 mm (AL < 26 group). (b) Eyes in AL ≥ 26 group were further divided into 26 ≤ AL < 28 group and AL ≥ 28 group. ^*∗*^NS Mann-Whitney *U* test.

**Figure 4:**
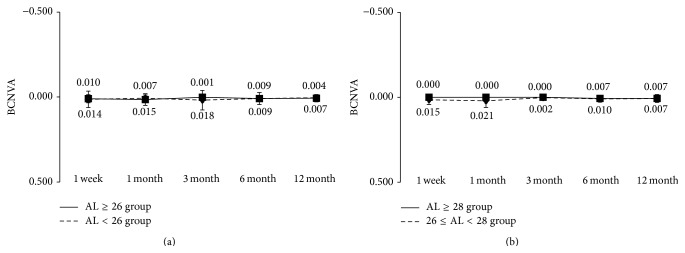
Best corrected near visual acuity during 12-month follow-up after implantation of multifocal intraocular lens. (a) Eyes were divided into axial length of 26 mm or longer (AL ≥ 26 group) and less than 26 mm (AL < 26 group). (b) Eyes in AL ≥ 26 group were further divided into 26 ≤ AL < 28 group and AL ≥ 28 group. ^*∗*^NS Mann-Whitney *U* test.

**Figure 5:**
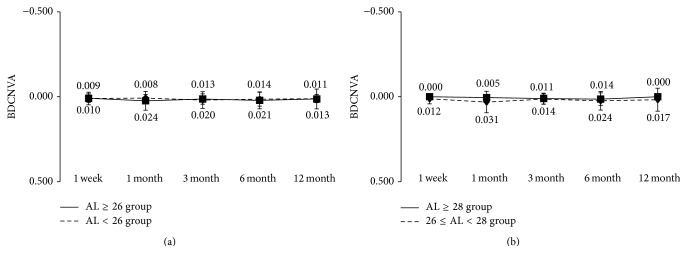
Best distance-corrected near visual acuity during 12-month follow-up after implantation of multifocal intraocular lens. (a) Eyes were divided into axial length of 26 mm or longer (AL ≥ 26 group) and less than 26 mm (AL < 26 group). (b) Eyes in AL ≥ 26 group were further divided into 26 ≤ AL < 28 group and AL ≥ 28 group. ^*∗*^NS Mann-Whitney *U* test.

Regarding near visual acuity, the mean uncorrected visual acuity at 12 months after surgery in the AL ≥ 26 group and the AL < 26 group was 0.06 ± 0.08 and 0.05 ± 0.09, respectively (Figure 3); the mean best corrected visual acuity was 0.01 ± 0.02 and 0.00 ± 0.02 (Figure 4); and the mean best distance-corrected visual acuity was 0.01 ± 0.06 and 0.01 ± 0.02 (Figure 5), also showing favorable outcome. During the observation period from 1 week to 12 months after surgery, no significant differences in uncorrected, corrected, and distance-corrected near visual acuity were observed between the two groups, similar to the results for distance visual acuity. In both groups, good visual acuity was achieved from early after surgery, with uncorrected, best corrected, and best distance-corrected visual acuity of 0.10 or above. We also divided the AL ≥ 26 group into a group with 26 ≤ AL < 28 mm and a group with ≥28 mm and conducted the same analysis. No significant differences were found between two groups (Figures 3(b), 4(b) and 5(b)).

### 3.2. Refractive Error

The postoperative refractive error is shown in Table 2. The postoperative absolute spherical equivalent was 0.29 ± 0.23 D in the AL ≥ 26 group and 0.35 ± 0.37 D in the AL < 26 group, with no significant different between two groups (*p* = 0.956). The proportion of eyes with postoperative spherical equivalent between +0.50 D and −0.50 D was 84.4% (27/32 eyes) in the AL ≥ 26 group and 78.1% (52/64 eyes) in the AL < 26 group, with no significant difference between two groups (*p* = 0.469, chi-squared test).

We also divided the AL ≥ 26 group into a group with 26 ≤ AL < 28 mm and a group with AL ≥ 28 mm and compared the refractive error. The postoperative absolute spherical equivalent was 0.29 ± 0.29 D in the AL ≥ 28 group and 0.29 ± 0.21 D in the 26 ≤ AL < 28 group, with no significant difference between the two groups (*p* = 0.869) (Table 2).

### 3.3. Depth of Focus

**Figure 6:**
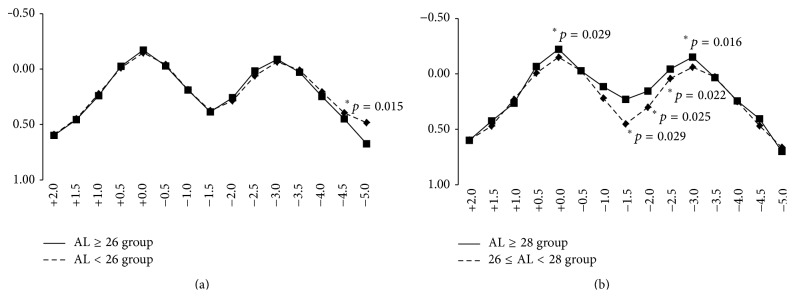
Defocus curves. (a) Eyes were divided into axial length of 26 mm or longer (AL ≥ 26 group) and less than 26 mm (AL < 26 group). Bimodal curves are shown with two peaks at spherical equivalent addition of 0.0 D and −3.0 D. Although a significant difference between two groups is observed at spherical equivalent addition of −5.0 D, no significant differences were found at the other values. (b) Eyes in AL ≥ 26 group were further divided into 26 ≤ AL < 28 group and AL ≥ 28 group. Bimodal curves are alsoobserved. Significant differences are detected at additional spherical equivalent of 0.0, −1.5, −2.0, −2.5, and −3.0 D. ^*∗*^Mann-Whitney *U* test.

In the AL ≥ 26 and AL < 26 groups, the defocus curves showed a bimodal distribution with two peaks at additional spherical equivalent of 0.0 and −3.0 D (Figure 6(a)). A significant difference between two groups was observed at spherical equivalent addition of −5.0 D, while no significant differences were observed at the other values. At additional spherical equivalent from −1.0 to −2.0 D, which is related to intermediate visual acuity, the vision in this range is lower than the distance and near vision.

We also divided the AL ≥ 26 group into a group with 26 ≤ AL < 28 mm and a group with AL ≥ 28 mm for comparison. Bimodal curves were observed in both groups. Significant differences were found at additional spherical equivalent of 0.0, −1.5, −2.0, −2.5, and −3.0 D (Figure 6(b)).

### 3.4. Contrast Sensitivity

**Figure 7:**
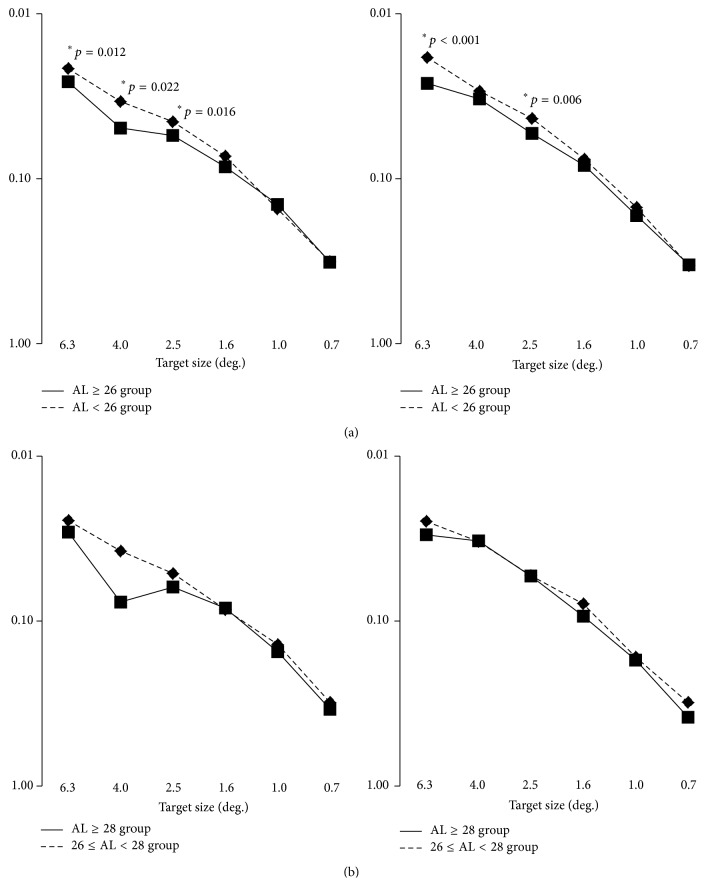
Contrast sensitivity at various target sizes. (a) Eyes were divided into axial length of 26 mm or longer (AL ≥ 26 group) and less than 26 mm (AL < 26 group). In the absence of glare, significant differences in contrast sensitivity are observed between two groups at target sizes of 6.3°, 4.0°, and 2.5°. In the presence of glare, significant differences are observed between two groups at target sizes of 6.3° and 2.5°. (b) Eyes in AL ≥ 26 group were further divided into 26 ≤ AL < 28 group and AL ≥ 28 group. No significant differences are observed. ^*∗*^Mann-Whitney *U* test.

Figure 7(a) shows the contrast measurements with glare (10000 cd/m^2^) and without glare in the AL ≥ 26 and AL < 26 groups. In the absence of glare, significant differences between the AL < 26 and AL < 26 groups were observed at target sizes of 6.3°, 4.0°, and 2.5°. Under glare condition, significant differences between two groups were observed at 6.3° and 2.5°.

When we divided the AL ≥ 26 group into a group with 26 ≤ AL < 28 mm and a group with AL ≥28 mm, no significant differences were found between two groups (Figure 7(b)).

### 3.5. Glare and Halo Disturbance

In both AL ≥ 26 and AL < 26 groups, none of the patients complained of severe glare or halo that impairs daily activities.

### 3.6. OCT Examination

The central subfield thickness was 266 *μ*m in the AL ≥ 26 group and 257 *μ*m in the AL < 26 group, with no significant difference between two groups (*p* = 0.336). In both groups, no abnormal findings in the macular region, including serous cystoid macular edema and myopic traction maculopathy, were observed.

### 3.7. Spectacle Use

After surgery, 19.0% (4 of 21 patients) in the AL ≥ 26 group and 17.5% (7 of 40 patients) in the AL < 26 group used spectacles after surgery, with no significant difference between two groups (*p* = 0.881, chi-squared test). In the AL ≥ 26 group, 3 patients used near vision spectacles, 1 used intermediate vision spectacles, and none used distance vision spectacles. Among the patients using near vision spectacles, one had postoperative spherical equivalent of +1.00 D and one had +0.50 D, while one had postoperative corneal astigmatism of +1.75 D. All three had uncorrected near visual acuity around 0.15. These patients had good corrected near visual acuity despite slightly poor uncorrected near visual acuity. In the AL < 26 group, 4 patients used distance vision spectacles, 3 patients used intermediate vision spectacles, and none used near vision spectacles. Among the patients who used distance vision spectacles, one had postoperative spherical equivalent of +1.00 D and one had −1.25 D, while one had postoperative corneal astigmatism of +1.50 D and one had +1.75 D. All four had uncorrected distance visual acuity around 0.15. These patients had good corrected distance visual acuity despite slightly inferior uncorrected distance visual acuity. However, in both groups, spectacle users wore the spectacles only when they had difficulties reading, and none of them used the spectacles all the time.

### 3.8. Rate of YAG Laser Treatment

In both AL ≥ 26 and AL < 26 groups, no patient underwent YAG laser treatment during 12 months after surgery.

### 3.9. Level of Satisfaction after Surgery

In both AL ≥ 26 and AL < 26 groups, none of the patients expressed dissatisfaction regarding postoperative visual function, and none of them desired to exchange the lens to monofocal IOL.

## 4. Discussion

Previous studies implanting multifocal IOL such as SN6AD3 and SN6AD1 in high myopic eyes have reported good distance and near visual function after surgery [4, 5]. Clinical evaluations of the multifocal ZMA00 and ZMB00 lenses have also shown good distance and near visual function after surgery [6–8], but postoperative outcome in high myopic eyes has not been reported. In the present study, we observed favorable postoperative visual functions also in high myopic eyes implanted with ZMA00 and ZMB00.

### 4.1. Visual Acuity

In this study, no significant differences in uncorrected and corrected visual acuity, both distance and near, were observed between the AL ≥ 26 group and AL < 26 group, and good visual acuity was achieved from early after surgery in both groups. In general, refractive error is known to be larger in high myopia [9, 10]. Previous studies evaluating other multifocal IOL reported less favorable uncorrected distance visual acuity in high myopia compared to low myopia [4, 5]. Our observation of no significant difference in uncorrected visual acuity between the higher and lower myopia groups may be explained by the finding of no significant difference in absolute spherical equivalent after surgery between two groups (*p* = 0.956) (Table 2). Furthermore, approximately 80% of the patients in both AL ≥ 26 and AL < 26 groups had postoperative spherical equivalent ranging from +0.50 to −0.50 D, with no significant difference between two groups. The reason for the above findings is that, even in eyes with long axial lengths, there was little error from the target refractive power, indicating high accuracy of IOL power estimation. We then analyzed whether the outcome differs depending on the IOL model. In the AL ≥ 26 group, 83.3% (15/18 eyes) using ZMA00 and 85.7% (12/14 eyes) using ZMB00 had postoperative spherical equivalent from +0.50 to −0.50 D, and the corresponding proportions in the AL < 26 group were 75.0% (27/36 eyes) using ZMA00 and 82.1% (23/28 eyes) using ZMB00, with no significant differences between two groups (*p* = 0.787, chi-squared test). These findings show that difference in axial length and difference in IOL model had no effect on the postoperative refraction. Previous reports recommended using an optical axial length measuring device together with the Haigis or SRK-T formula for calculating lens power for high myopic eyes to reduce the postoperative refraction error [10, 11]. In our hospital, we also use the IOLMaster optical axial length measuring device to measure axial length and determine the target refraction and lens power using the SRK-T formula. Furthermore, Bang et al. [10] reported that longer axial length is associated with a greater tendency of hyperopia after surgery and recommended target refraction of −0.25 to −0.75 D for axial lengths of 27.00 to 29.07 mm and −0.50 to −1.00 D for axial lengths of 29.07 to 30.62 mm for those aiming for emmetropia after surgery. In the AL ≥ 26 group, we also selected the target refractive power considering the tendency of hyperopia after surgery. This strategy probably results in the small deviation in postoperative refractive power even in high myopic eyes and the absence of significant difference in postoperative absolute spherical equivalent between the higher and lower myopic groups.

Since the AL ≥ 26 group had axial lengths ranging from 26 to 29 mm, we examined whether the degree of myopia in this group influences the results. By dividing this group into a group with 26 ≤ AL < 28 mm and a group with AL ≥ 28 mm, our analysis showed no significant differences in visual acuity (except near corrected distance visual acuity at 3 months after surgery), refractive error, and depth of focus between the two groups, indicating the validity of analyzing AL ≥ 26 as a group.

We compared the 6-month postoperative visual acuity of the present study to those of SN6AD3 with the same near addition power for high myopic eyes (Table 3). According to Wang et al. [4], the uncorrected distance visual acuity (UDVA) was 0.18 ± 0.12, best corrected visual acuity (BCDVA) was 0.05 ± 0.05, uncorrected near visual acuity (UNVA) was 0.10 ± 0.07, and best distance-corrected near visual acuity (BDCNVA) was 0.07 ± 0.08. In the report of Alfonso et al. [5], the UDVA was 0.18 ± 0.20, BCDVA was 0.05 ± 0.09, UNVA was 0.09 ± 0.10, and BDCNVA was 0.07 ± 0.08. Compared with the above reports, ZMA00 and ZMB00 provided the same or better visual acuity.

### 4.2. Contrast Sensitivity

Due to the optical design, contrast sensitivity may decrease with the use of diffractive multifocal IOL. Previous clinical studies of other multifocal IOL also indicated lower contrast sensitivity with multifocal IOL [11–13]. In the present study, irrespective of the presence or absence of glare, the contrast sensitivity in the low spatial frequency range was lower in the AL ≥ 26 group than in the AL < 26 group. However, we observed no waxy vision and other serious symptoms presumably related to low contrast sensitivity. Thus, the lowered contrast sensitivity observed in the present study is considered to have almost no effect on visual function. In the report of Alfonso et al. [12], compared to low myopic eyes, high myopic eyes showed lower contrast sensitivity mainly at higher spatial frequencies under photopic condition (85 cd/m^2^) and lower contrast sensitivity at all spatial frequencies under mesopic condition (5 cd/m^2^).

### 4.3. Patient Satisfaction after Surgery

Almost all patients with high myopia wear spectacles or contact lenses. Even with good corrected visual acuity, some patients use spectacles or contact lenses in a low correction state and may not achieve adequate visual acuity for daily living. For myopic eyes, cataract surgery may improve the myopia. Therefore, cataract surgery not only treats cataract but also has a role in correcting refraction. After surgery, many patients are delighted that they are relieved from or can reduce the frequency of using spectacles or contact lenses that they have worn over long years. In the present study, almost all patients with axial length ≥26 mm wore spectacles or contact lenses before surgery. After surgery, approximately 81% of the patients with axial lengths ≥26 mm were relieved completely from using spectacles or contact lenses. For the remaining 19%, although their uncorrected distance visual acuity was slightly poor, vision had improved compared to before surgery, and the patients used spectacles only when they had difficulties reading. They were satisfied with multifocal IOL implantation for the reduced dependence on spectacles. It is noteworthy that three of seven patients who wore spectacles after surgery had postoperative corneal astigmatism of +1.50 to +1.75 D, indicating that, other than residual myopia or hyperopia, astigmatism is a common cause of poor postoperative uncorrected visual acuity.

In patients with bilateral high myopia undergoing cataract in one eye, the problem of anisometropia arises. With anisometropia of 2.00 to 2.50 D, a difference of 5% occurs in the retinal image and this range is considered to be the limit of binocular vision [14]. However, the change in retinal image is smaller when using contact lenses than when using spectacles. Therefore, when performing cataract surgery on one of two high myopic eyes, it is desirable to implant a multifocal IOL that corrects postoperative refraction to 0 D and prescribe contact lens for the contralateral eye. In the present study, 10 patients in the AL ≥ 26 group underwent cataract surgery in one eye only. All the patients had experience of using contact lenses and there was no resistance to using contact lenses in the contralateral eye. Among patients who had multifocal IOL implantation with postoperative refraction targeted at 0 D in one eye and wore distance-corrected contact lens in the contralateral eye, none of them complained of anisometropia. There was no case of IOL extraction due to difference in visual function between two eyes after surgery.

However, since visual function impairment in high myopic eyes may be caused by factors other than cataract, such as high myopia-related retinopathy and optic neuropathy, we perform ophthalmoscopic examination and OCT before surgery to evaluate macular function. Cases with abnormalities are contraindicated for multifocal IOL implantation. Appropriate selection of cases indicated for multifocal IOL implantation was a major factor that yielded good postoperative visual function and high level of patient satisfaction in the present study.

## 5. Conclusion

When performing multifocal IOL implantation in eyes with long axial lengths, it is possible to achieve highly accurate postoperative refractive power by determining axial length using an optical axial length measuring device and selecting IOL power with appropriate correction according to the axial length. Furthermore, to obtain good postoperative visual function, detailed preoperative evaluation of the optic nerve and macular region is important. By selecting cases carefully as described above, it is possible to achieve good uncorrected distance and near visual acuity by implantation of multifocal IOL in eyes with long axial lengths. Even in patients with high myopia who required constant use of spectacles or contact lenses, the frequency of spectacle use decreased and a high level of satisfaction was obtained. The present study demonstrates that multifocal IOL implantation is useful for eyes with long axial lengths.

## Figures and Tables

**Table 1:** Demographic and clinical characteristics of patients stratified into axial length of 26 or above (AL ≥ 26 group) and axial length below 26 mm (AL < 26 group).

	AL ≥ 26 group	AL < 26 group	*p* value
Number of eyes	32	64	
ZMA00	18	36	
ZMB00	14	28	
Age (y) mean ± SD (range)	57.0 ± 10.9	59.1 ± 8.2	0.703
	(40–71)	(41–69)	
Male/female	15/6	13/27	
Axial length (mm)	27.38	24.13	<0.001
Mean (range)	(26.06–29.24)	(22.00–25.95)	

**Table 2:** Postoperative refractive data. (A) Patients were stratified into axial length of 26 or above (AL ≥ 26 group) and below 26 mm (AL < 26 group). (B) Eyes in AL ≥ 26 group were further divided into 26 ≤ AL < 28 group and AL ≥ 28 group.

	A			B		
	AL ≥ 26 group	AL < 26 group	*p* value	26 ≤ AL < 28 group	AL ≥ 28 group	*p* value
Postoperative spherical equivalent (D)	0.14 ± 0.35 (−0.50 to +0.75)	−0.25 ± 0.44 (−1.50 to +1.00)	<0.001	0.18 ± 0.38 (−0.50 to +0.75)	−0.12 ± 0.34 (−0.50 to +0.75)	0.742

Postoperative absolute spherical equivalent (D)	0.29 ± 0.23 (0.00 to +0.75)	0.35 ± 0.37 (0.00 to +1.50)	0.956	0.29 ± 0.29 (0.00 to +0.75)	0.29 ± 0.21 (0.00 to +0.75)	0.869

Postoperative refractive error (D)	0.29 ± 0.37 (−0.60 to +1.04)	−0.23 ± 0.36 (−1.25 to +0.52)	<0.001	0.44 ± 0.23 (+0.24 to +0.86)	−0.25 ± 0.40 (−0.60 to +1.04)	0.232

Postoperative absolute refractive error (D)	0.39 ± 0.27 (+0.02 to +1.04)	0.30 ± 0.30 (0.00 to +1.25)	0.068	0.44 ± 0.23 (+0.24 to +0.86)	0.37 ± 0.28 (0.00 to +1.04)	0.477

Postoperative corneal astigmatism (D)	0.66 ± 0.44 (+0.00 to +1.75)	0.64 ± 0.42 (+0.00 to +1.75)	0.730	0.83 ± 0.33 (+0.25 to +1.25)	0.60 ± 0.47 (+0.00 to +1.75)	0.112

Data are expressed as mean ± SD (range).

**Table 3:** Distance and near visual acuity following implantation of multifocal intraocular lenses: the present study and those of Wang et al. [4] and Alfonso et al. [5].

	This report ZMA00, ZMB00	Wang et al. [4] SN6AD3	Alfonso et al. [5] SN6AD3
UDVA	−0.04 ± 0.13	0.18 ± 0.12	0.18 ± 0.20
BCDVA	−0.15 ± 0.06	0.05 ± 0.05	0.05 ± 0.09
UNVA (30 cm)	0.07 ± 0.10	0.10 ± 0.07	0.09 ± 0.10
BDCNVA (30 cm)	0.02 ± 0.05	0.07 ± 0.08	0.07 ± 0.08

UDVA, uncorrected distant visual acuity; BCDVA, best corrected visual acuity; UNVA, uncorrected near visual acuity; BDCNVA, best distance-corrected near visual acuity.
